# Amino acid homorepeats in tape measure proteins correlate with bacteriophage tail length

**DOI:** 10.1099/jgv.0.002341

**Published:** 2026-09-17

**Authors:** Antonio Moreno-Rodríguez, Antonio J. Pérez-Pulido, Pablo Mier

**Affiliations:** 1 Andalusian Centre for Developmental Biology (CABD, UPO-CSIC-JA), Faculty of Experimental Sciences (Genetics Area), Universidad Pablo de Olavide, 41089 Dos Hermanas, Spain

**Keywords:** homorepeat, phage, phage tail, tandem repeat, tape measure protein

## Abstract

The tape measure protein (TMP) dictates the tail length of tailed bacteriophages. However, the sequence features that drive the process of length fine-tuning have not yet been described. Tandem repeats (TRs), stretches of aas organized as multiple adjacent copies of the same or very similar sequence motif, are contributors to TMP length variation. Homorepeats (polyX regions) are a specific type of TRs composed of stretches of identical aas, forming low-complexity regions that contribute to protein structural flexibility and dynamics. Here, we performed a large-scale analysis of polyX regions across 12 million phage proteins to test whether these homorepeats help explain the variable length of flexible phage tails. PolyX tracts were detected in 41% of all phage proteins and in 95.5% of TMPs, with polyA, polyG, polyS and polyT dominating the composition. Furthermore, the number of polyX tracts in TMPs scaled directly with protein length. About half of the TMPs have both polyX and TRs (23,469 out of 45,500 TMPs), but nearly half of the polyX-containing TMPs lacked any TRs (19,999 out of 43,468 TMPs), showing that polyX are independent features rather than by-products of larger repeats. Morphological comparisons showed that phages with long and flexible tails (Siphovirus morphotype) combine polyX and TRs, whereas shorter or contractile-tailed phages rely mostly on polyX. Last, the total aa content in polyX regions correlated significantly with experimentally measured tail lengths (*R*²=0.811, *P*=1.56×10^−^⁴). Our results identify polyX tracts as an important and tunable component of TMPs that enable precise control of phage tail length.

## Data availability

The data underlying this article are available in the article and in its online supplementary material.

## Introduction

Bacteriophages (abbreviated as phages) are one of the most prevalent and ubiquitous organisms in the world [1]. They are viruses that specifically infect bacteria and are among the most abundant and genetically diverse biological entities on Earth, exerting immense control over microbial ecology and evolution. Phages shape bacterial population dynamics, mediate horizontal gene transfer and influence global biogeochemical cycles [2].

The infection process involves several steps. One of the most critical steps is the entry of the phage’s genetic material into the bacterial cell. This step can occur through various mechanisms: icosahedral phages can form a tube through a conformational change in their structure, which penetrates the bacterial cell wall and injects the genetic material [3]; filamentous phages interact with a surface structure of bacteria, the pili, unpacking the genetic material into the inner membrane [4]; phages with a lipid membrane fuse with the bacterium’s membrane [5]. On the other hand, every successful infection by tailed phages (classified as Caudovirales) relies on a highly specialized nanomachine, a contractile or non-contractile tail that binds the bacterial surface and injects the viral genome through the cell envelope. Beyond a structural appendage, the tail determines host range, infection efficiency and viral morphology, with its length, stiffness and modular organization dictating virion geometry and DNA delivery mechanics. Central to this is the tape measure protein (TMP), a long α-helical element spanning the tail tube to precisely set its length [6, 7]. Experimental truncations and insertions in TMP genes produce tails that scale linearly with TMP size, directly linking sequence length to virion architecture [8].

Using this morphological characteristic, the Caudovirales order was classified into three families: *Podoviridae*, short and non-contractile tails; *Siphoviridae*, long and non-contractile tails; and *Myoviridae*, complex and contractile tails [9]. Although this classification has become obsolete (it only considers tail length, a feature common across phylogenetically unrelated phages), it still helps group phages sharing structural and infection-related characteristics [10, 11]. Previous studies have associated TMP size and tail length only in Siphovirus [12] and Myovirus morphotypes [13]. To the best of our knowledge, there is no information relating Podovirus morphotype phages with TMP.

In some phage TMPs, tandem repeats (TRs) are known to be contributors to the protein’s overall length variation [14]. Homorepeats, or polyX regions, are considered the simplest type of TR, being a special case in which the repeated unit has a length of one aa; they are low-complexity regions composed of stretches of identical aa (the X in polyX) [15]. However, depending on the threshold used to detect them, they can contain a limited amount of non-identical aas (‘impurities’). The most used threshold to look for polyX regions is ≥4/6, that is there must be four identical aas in a local region of six residues to consider it as a polyX [16]. They can be found in all domains of life [17] and have been linked to structural flexibility, liquid–liquid phase separation and rapid evolutionary change [18–20]. Both TR and polyX length variation are thought to arise via the same underlying mechanism, replication slippage [21, 22], which allows small insertions or deletions that modulate protein length without disrupting the reading frame.

PolyX regions are well characterized in eukaryotes [23] but remain poorly studied in prokaryotes due to their lower prevalence [24]. A recent study expanded the polyX knowledge to giant viruses [25]. It found homorepeats in this group to be ubiquitous and enriched in structural and host-interaction proteins. That work suggested that repeat-rich viral proteins use low-complexity regions to mediate flexible structural assembly and adaptive plasticity. However, whether smaller viruses such as phages use similar principles remains unknown. Despite the structural complexity and evolutionary dynamism of phage tails, no systematic analysis has yet examined the occurrence or role of low-complexity regions in them.

Here, we address this gap by doing a large-scale survey of homorepeats across all phage proteins in the IMG/VR dataset from the PhageScope database. We quantify their prevalence and aa composition, evaluate their enrichment across functional categories and focus on the subset of TMPs, key structural determinants of tail length. We further compare polyX organization with TRs to test whether these motifs arise as independent features or as parts of longer repetitive regions. Furthermore, we analysed the TMP sequences of phages with experimentally measured tail lengths to test the hypothesis that tail length is related to the presence of polyX or TRs.

## Methods

### Data retrieval

Phage protein datasets were obtained from the IMG/VR section of the PhageScope v1.3 database [26]. The downloaded data contained 12,036,448 proteins from 177,361 phages. The corresponding metadata files (‘Phage Meta Data’ and ‘Annotated Protein Meta Data’) were downloaded to provide functional and taxonomic annotations. Protein annotations were identified using case-insensitive searches within the annotation field.

### PolyX and TR search

PolyX regions were detected in all phage proteins using the standalone version of the polyX2 tool [27], with a default threshold of ≥4/6 (implying a minimum number of four identical residues in a region of six aas). Each polyX-containing protein was mapped to its corresponding annotation from the IMG/VR metadata. A non-redundant list of proteins containing at least one polyX was generated.

The subset of proteins annotated as tape measure was extracted from the IMG/VR dataset. Their polyX tracts were retrieved and analysed separately (File S1, available in the online Supplementary Material). Distances between consecutive polyX tracts within the same protein were computed based on their start coordinates.

TRs were identified in TMPs using DetectRepeats from the DECIPHER R package [28] (File S2). We then categorized the TR-containing proteins based on whether they also include polyX regions.

Statistical comparisons between groups were performed using two-sided Wilcoxon rank-sum tests; *P*-values were adjusted for multiple testing using the Benjamini–Hochberg (BH) procedure. To assess the relationship between the number of polyX regions and TMP length, TMPs were first grouped by their number of polyX regions (including TMPs with no polyX). Then, the median protein length was calculated for each group, and finally, a linear model was fitted to these median values for groups with 0–25 polyX per protein. Groups with more than 25 polyX per protein were excluded due to their low sample size.

### Functional and taxonomic analyses

Protein functions were grouped using the IMG/VR annotation field, and their prevalence among polyX-containing proteins was computed. The ten most frequent annotations in the complete dataset (all phage proteins, background) and in the polyX subset were compared. Enrichment was calculated as the ratio between the two proportions. Phage taxonomy was retrieved from IMG/VR metadata.

Phage taxonomy was retrieved by classifying their proteins using hmmscan (HMMER v.3.1b2 [29]) with three Hidden Markov Model (HMM) profile databases, each related to a morphological family (Myovirus, Podovirus or Siphovirus). These profiles are based on phage Viral Orthologous Groups (pVOGs), which collect the characteristic proteins of each taxon [30].

The results for each phage were then processed, retaining only those hits with an e-value of less than 1×10^−5^ and a score of more than 30. Taxa supported by fewer than two hits were discarded.

To standardize the contribution of each protein to the definitive classification of the virus, the HMMER score was normalized by the number of valid hits for each phage. Additionally, a taxon score was calculated for each of the three families using this formula:


$$taxonscore=\frac{normalized\sum \cdot {\left(Nhits\right)}^{0.7}}{{\left(Nprofiles\right)}^{0.6}}$$

*N*hits is the number of valid HMM hits assigned to that taxon, and *N*profiles is the number of pVOG profiles used for that taxon. For each phage, the chosen family was the one with the highest taxon score. To avoid false positives, an ambiguity ratio was defined, in which if the ratio of the greatest taxon score to the second-greatest taxon score was less than 1.1, the phage was considered ambiguous. As a positive control for this procedure, known phages described in the literature and empirically verified for morphology were classified (File S3).

### Validation with phages of known tail length

Phage tail-length measurements were manually compiled from the literature (File S3). TMPs of those phages were downloaded from UniProtKB release 2025_04 [31] and analysed for polyX and TRs using the same procedures described above.

## Results

### Global characterization and functional enrichment of polyX regions in phage proteins

**Fig. 1.**
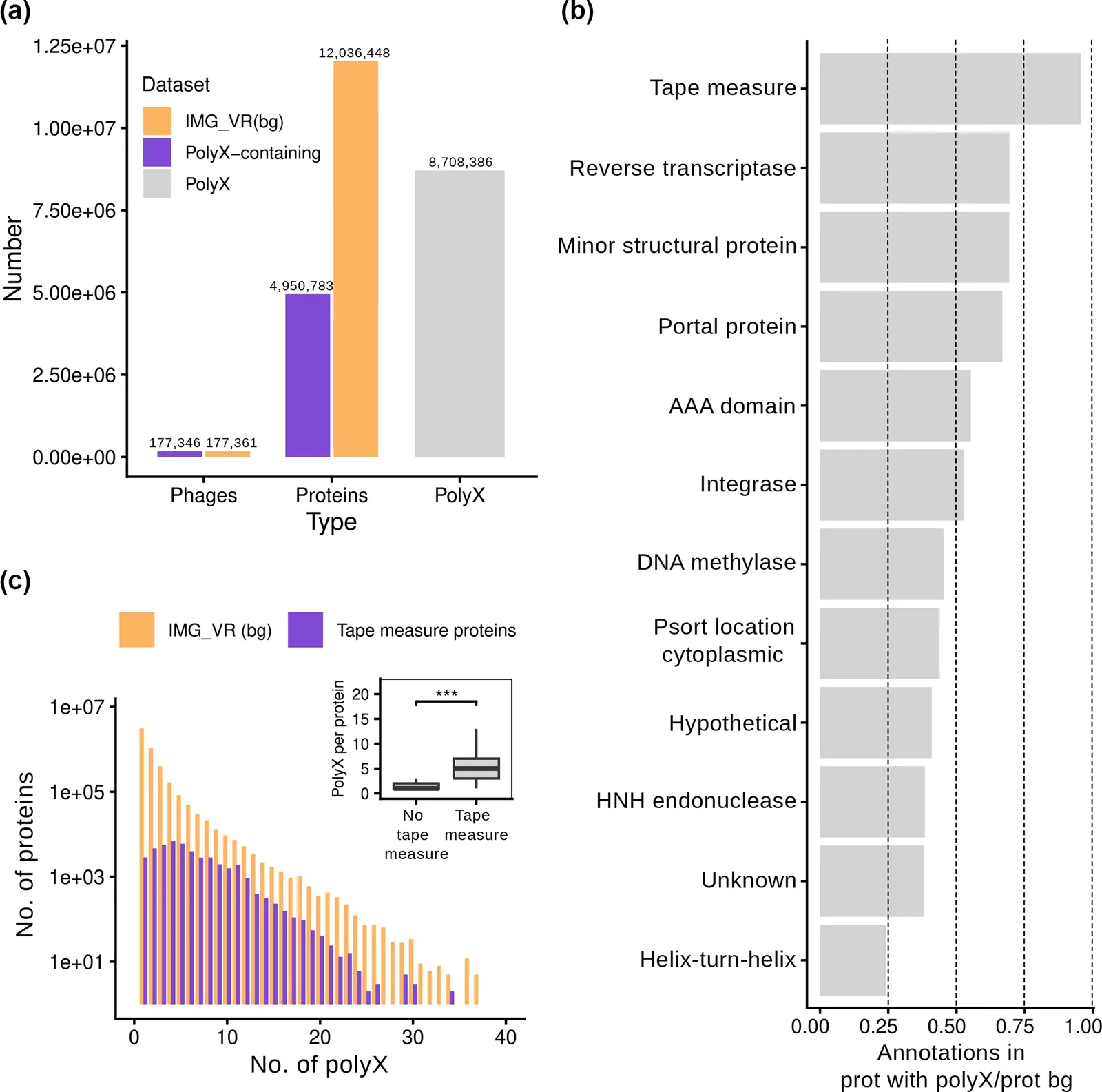
Characterization of polyX regions in phage proteins. (**a**) Number of phages, proteins and polyX in the background dataset (IMG/VR) and in polyX-containing sequences; polyX-containing proteins represent 41.1% of the total protein count in the background dataset. (**b**) Enrichment ratios for functional annotations in phage proteins with polyX compared to all phage proteins; the ten most enriched annotations from each dataset (twelve unique in total) are shown. (**c**) Log_10_ protein count and number of polyX regions in the background dataset (IMG/VR) and in proteins annotated as tape measure. Inset shows the number of polyX regions per protein and whether or not they are annotated as TMPs. Statistical comparisons between groups were performed using Wilcoxon rank-sum tests (*** corresponds to *P*-value <0.001 after BH adjustment).

We evaluated the prevalence of polyX regions in aa sequences of phages. For that, we retrieved 12,036,448 protein sequences from 177,361 different phages from the IMG/VR section of the PhageScope database. This comprehensive dataset of phage proteins served as the background dataset for our study. We searched for polyX regions in the background dataset using a lax threshold of at least four identical residues in a local region of six aas and identified 8,708,386 polyX tracts in 4,950,783 proteins (Fig. 1a). PolyX regions appeared as a highly common feature in phage proteins, with ~41.1% of all proteins in the dataset containing at least one polyX. For the sake of comparison, a similar computation with the human proteome results in 81.6% of its proteins having at least one polyX (16,872 out of 20,659 proteins); for the proteome of *Escherichia coli K12*, this number is reduced to 56.2% (2,474 out of 4,402 proteins). Those phage polyX-containing proteins represent 177,346 phages, showing a nearly ubiquitous presence across the phage population analysed (99.99% of phages have at least one polyX-containing protein).

We next assessed the functional roles of the polyX-containing proteins by comparing the prevalence of functional annotations in that subset to the background dataset. We merged the two sets of the ten most prevalent annotations per dataset, for a total of twelve distinct annotations. The strongest enrichment was found for TMPs (Fig. 1b). In IMG/VR-labelled TMPs, 43,468/45,500 (95.53%) contained at least one polyX region. The total number of polyX regions located in this dataset is 240,964 (File S1). We also found a high enrichment for annotations ‘reverse transcriptase’, ‘minor structural protein’ and ‘portal protein’, further suggesting a functional significance of polyX regions within structural components of the phage.

Given the strong enrichment observed for TMPs, we focus hereinafter on TMPs. The distribution of the number of polyX regions per protein was then compared between the subset of TMPs and the overall background (Fig. 1c). The background protein distribution shows a rapid decay, indicating that the majority of proteins containing polyX have only one or two regions. In contrast, the distribution for TMPs is more uniform up to ten polyX per protein. This relative enrichment indicates that, compared to the general phage protein database, TMPs contain more polyX tracts.

### Characterization of polyX regions in TMPs

Following the enrichment of polyX regions in TMPs, we examined their organization, spacing and aa composition. This analysis covered 43,468 TMPs containing 240,964 polyX regions, with a mean length of 6.59 aas (File S1).

**Fig. 2.**
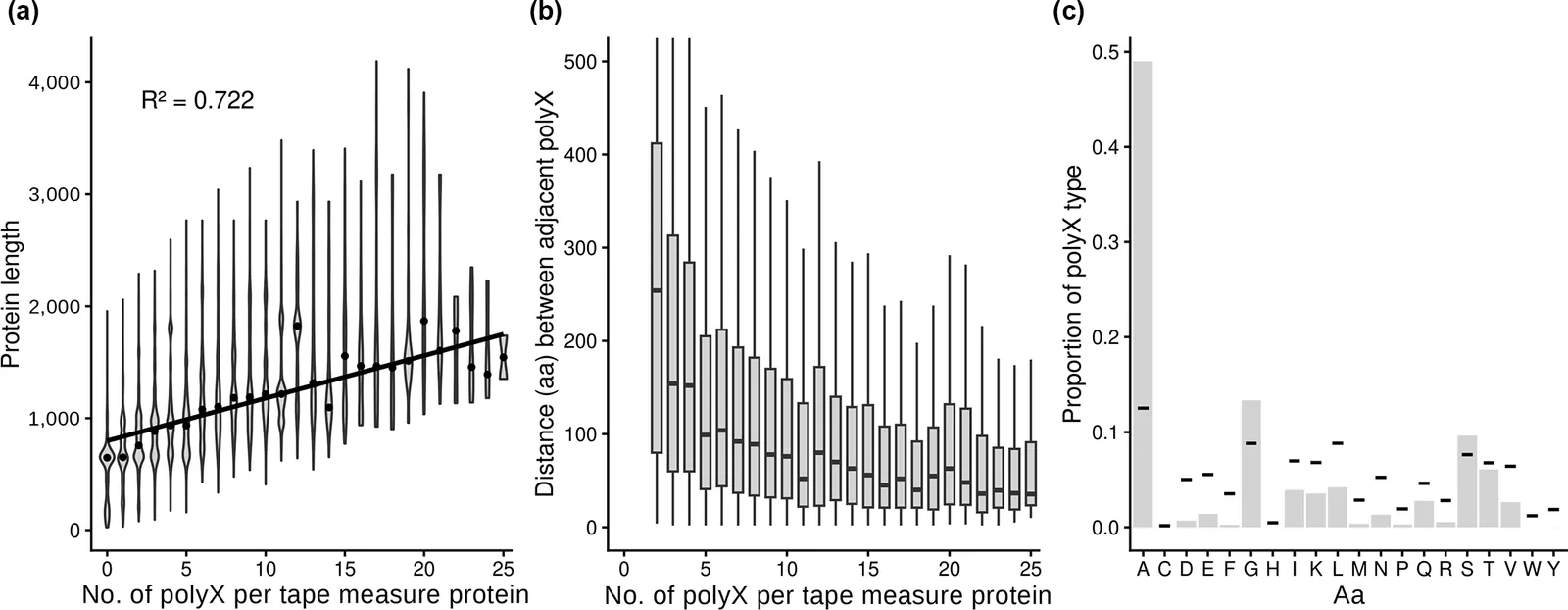
Characterization of polyX regions in TMPs. (**a**) Distribution of TMP lengths by number of polyX regions; regression line calculated with the median values. (**b**) Distance (aas) between adjacent polyX in TMPs with more than one polyX. (**c**) Proportion of each polyX type (e.g. proportion of all polyX regions that are polyA) across all polyX regions in TMPs (bars). Black horizontal lines indicate, for comparison, the overall frequency of each corresponding aa among all residues in the TMP set.

We first assessed the relationship between the number of polyX per protein and the overall protein length (Fig. 2a). Our analysis showed a positive correlation (*R*^2^=0.722, *P*=3.97×10^−^⁸), in which the presence of more polyX regions is associated with greater protein length. The median protein length increases from ~650 aas in proteins with 1 or 2 polyX to 1,868 aas for those with 20 polyX. This trend suggests that the accumulation of polyX regions leads to protein elongation, either because larger proteins provide more insertion sites for these low-complexity regions or because they themselves extend the protein.

Some TMPs are known to contain TRs that contribute to their length [14]. If polyX regions were exclusively and uniformly embedded within TRs, we would expect consistent spacing between them. Thus, we studied the distance between polyX within proteins with more than one of these regions. We found that the distance was shorter as the number of polyX increased (Fig. 2b); therefore, the distances between adjacent polyX tracts varied widely. The broad and irregular distribution of distances between polyX regions within proteins indicates that polyX regions occur as independent sequence motifs rather than contiguous units of repeats.

Finally, we examined the overall aa composition of all 240,964 polyX regions found in the TMP subset (Fig. 2c). Results show a strong overrepresentation of specific aas. PolyA regions are by far the most abundant polyX type (49% of all polyX regions), even though alanines account only for 12.52% of the aas in the set of TMPs. Following polyA, the most prevalent polyX types are polyG, polyS and polyT, which account for ~13%, 10% and 6% of the total, respectively. Together, polyA, polyG, polyS and polyT constitute nearly 80% of all polyX regions in TMPs. These four aas (A, G, S and T) are characterized by small side chains and give flexibility to the polypeptide chain. Moreover, alanine stretches have been linked to transcriptional slippage [32], making them perfect candidates to easily fine-tune phage tail length from an evolutionary perspective.

### Relationship between polyX regions and TRs in TMPs

We have so far established that polyX regions are highly enriched in TMPs and that their distribution suggests they are not exclusively components of larger, regular repeats. Given that TRs have previously been associated with variable TMP length [14] and that polyX are ultimately TRs with a unit length equal to one, we decided to compare the prevalence and co-occurrence of polyX and TRs within the 45,500 TMPs in our dataset.

**Fig. 3.**
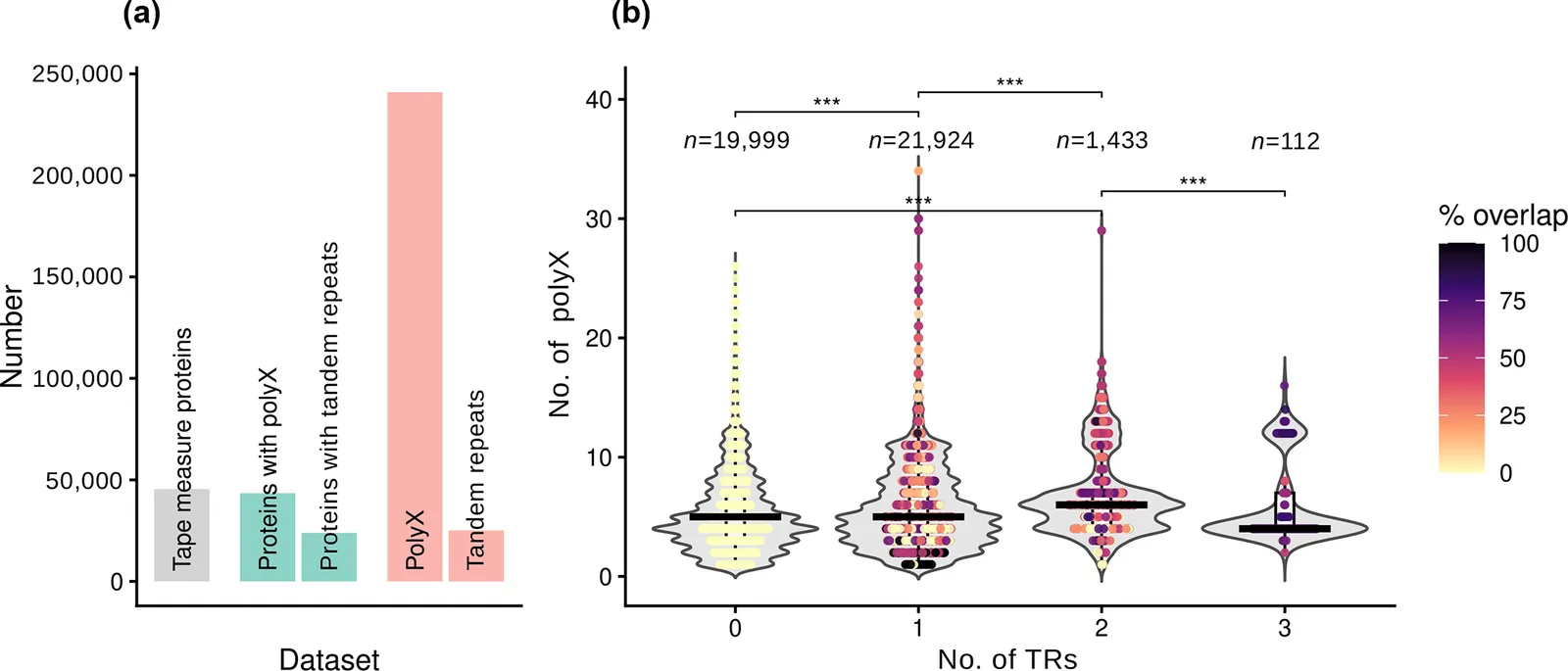
Comparison of polyX and TR-containing proteins in TMPs. (**a**) Number of proteins or regions per dataset. (**b**) Distribution of the percentage of polyX regions that overlap TRs, stratified by the number of TRs and polyX per protein. Statistical comparisons between groups were performed with two-sided Wilcoxon rank-sum tests; *P*-values were adjusted for multiple testing using the BH procedure (*** corresponds to *P*-value <0.001 after BH adjustment).

We identified TRs in TMPs. Among the 45,500 TMPs analysed, 23,873 (52.47% of the total) contain TRs, whereas 43,468 (95.53%) contain polyX regions (Fig. 3a). A total of 23,469 proteins have both repeat types, while only 404 proteins have TRs but no polyX. Interestingly, a large subset of polyX-containing TMPs do not have any detectable TRs (19,999 proteins or 46.0% of the polyX-containing TMP set). These results show that polyX regions are much more common than TRs in phage TMPs, further proving that polyX enrichment is a frequent and independent feature rather than a by-product of TR organization.

Proteins with more TRs have significantly more polyX regions (Fig. 3b). For each TMP with at least one polyX, we computed the percentage of that protein’s polyX regions that overlap a TR; these per-protein percentages are plotted for proteins grouped by their TR count. The proportion of overlapping polyX regions increases significantly with the number of TRs and the number of polyX per protein, suggesting that proteins with more repeats tend to integrate polyX within TRs. Conversely, proteins with few polyX regions show lower overlap, confirming that many polyX occur as isolated sequence motifs.

### Tail morphotypes reflect distinct repeat profiles in TMPs

Taxonomic information in the PhageScope database was not informative for our study, due to the lack of information on tail morphology. Of the 177,361 phages used as input, 90.8% were annotated as Caudoviricetes, which comprises tailed phages. TMPs belong to phages enriched in this taxonomic group (99.44%); however, due to the absent granularity of this taxonomy, no further conclusions can be obtained from this result.

**Fig. 4.**
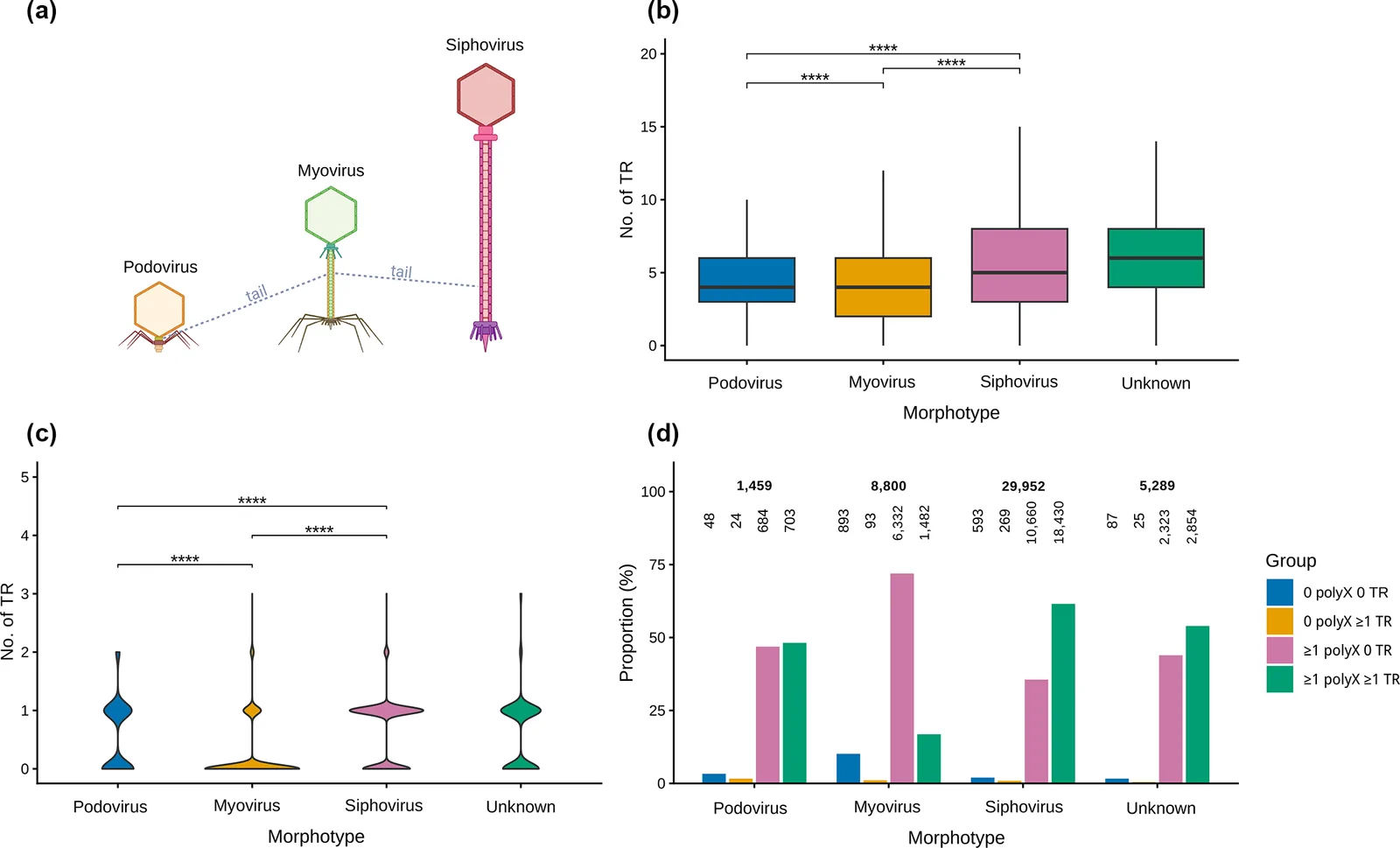
Analysis of TMPs across morphotypes. Results are shown as within-group proportions for Podovirus (*n*=1,459), Myovirus (*n*=8,800), Siphovirus (*n*=29,952) and Unknown (*n*=5,289). (**a**) Graphical representation of morphotypes used in the study. (**b**) Proportion of TMPs based on the number of polyX sequences. (**c**) Proportion of TMPs based on the number of TRs. (**d**) Proportion of TMPs based on the co-occurrence of polyX sequences and TRs. Statistical comparisons between groups were performed with two-sided Wilcoxon rank-sum tests; *P*-values were adjusted for multiple testing using the BH procedure (*** corresponds to *P*-value <0.001 after BH adjustment).

Phages do, however, have different tail sizes (Fig. 4a), so we could expect different TMP lengths in them. We classified each phage according to its protein hits: the morphotype most represented by its proteins, after normalization and calculation of a score (see Methods), was the morphotype assigned to the phage. We classified the phages with at least one TMP in three morphotype groups: Podovirus (*n*=1,459), Myovirus (*n*=8,800) and Siphovirus (*n*=29,952), which are known to correspond to short non-contractile, long complex contractile and long flexible non-contractile tails, respectively (File S4). We also included an ‘Unknown’ group (*n*=5,289). The unequal number of TMPs per group reflects the known absence of this protein in many Podovirus [33]. Taking into account the complete set of 177,361 phages, the number of phages with at least one TMP is also unbalanced between morphotypes: 1,459/22,880 Podovirus (6.38%), 8,800/34,113 Myovirus (25.80%) and 29,952/88,250 Siphovirus (33.94%). This proportion reflects a putative incomplete annotation in Siphovirus morphotype phages in the IMG/VR dataset, as most Siphovirus are expected to contain a TMP [34]. Recovering these likely unannotated TMPs (e.g. through positional or structural inference within the tail gene cluster) was beyond the scope of this study but represents a valuable direction for future work.

The distribution of the number of polyX regions per TMP in each morphotype group shows that TMPs without polyX regions are rare across all morphotypes, as expected because of our previously described finding that 95.53% of TMPs have at least one polyX (Fig. 4b). The distributions are similar for phages classified as Podovirus and Myovirus. In contrast, Siphovirus phages tend to have TMPs with a high number of polyX regions. This partially supports the hypothesis that a higher polyX count correlates with the longer tails found in Siphovirus.

This is complemented by the analysis of TRs (Fig. 4c). While roughly half of Podovirus TMPs have at least one TR, Myovirus TMPs are characterized by not having any TR. On the other hand, more than half of Siphovirus TMPs do have one or more TRs. The co-occurrence analysis synthesizes these findings (Fig. 4d). The pattern for Myovirus shows that TMPs are dominated by having only polyX (≥1 polyX and 0 TR). They seem to achieve their long tail lengths primarily using a high number of polyX regions in TMPs rather than through TRs. Siphovirus and Podovirus TMPs are both split, with TMPs containing either only polyX or a combination of both polyX and TRs. Across all morphologies, TMPs containing only TRs are negligible. These results establish polyX regions as the fundamental repeat feature in TMPs, acting either as the sole repeat mechanism or in combination with TRs.

### PolyX content correlates with experimentally measured phage tail length

To validate the hypothesis that polyX regions on TMPs are involved in determining phage tail length, we manually compiled an independent dataset of phages for which tail lengths have been experimentally measured and reported in the literature (File S3). While we found in the literature cases of Podovirus with an experimentally measured tail (29 nm in phage T7 [35], 38 nm in phage phi29 [36] and 40 nm in phage p22 [37]), none of them has a TMP. This prevents us from testing whether polyX content correlates with tail size in short-tailed phages within this experimentally validated dataset. This is a separate issue from the morphotype classification in the previous section, where Podovirus phages are still represented. That classification is based on HMM profile hits against phage proteins and does not require either an experimental tail-length measurement or the presence of an annotated TMP.

We analysed the TMPs from those phages using the same polyX and TRs detection methods used above. While all the identified TMPs from this dataset contained polyX regions, only two of them were found to also contain a TR (TMPs from phages SPP1 and P1; File S3). This observation supports our previous finding that polyX regions are a prevalent feature in TM proteins, often existing independently of TRs.

**Fig. 5.**
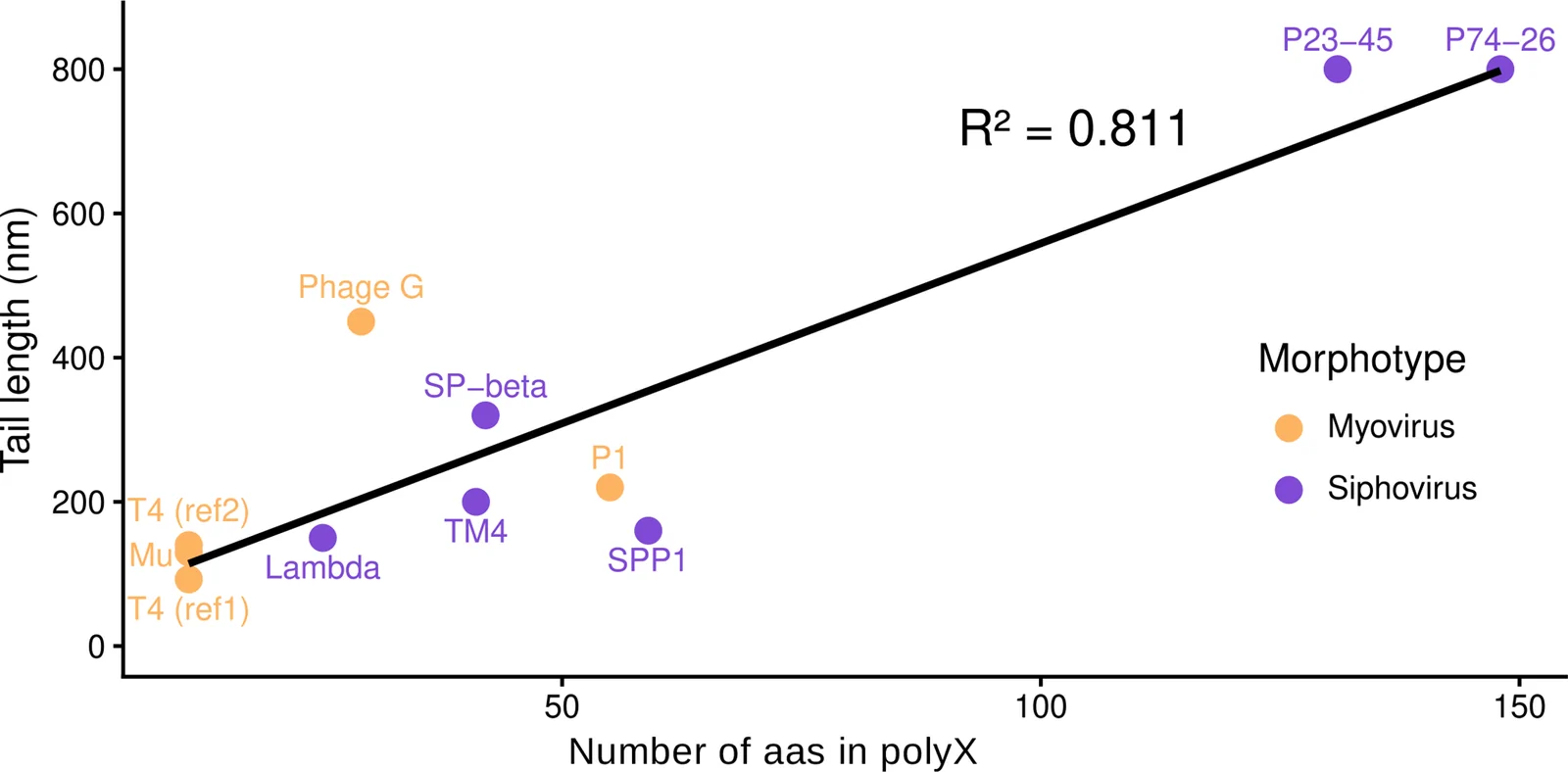
Relationship between polyX regions in TMPs and experimentally measured phage tail length. Phage tail length (nm) versus total number of aas in polyX regions. A linear regression line is shown, with goodness-of-fit *R*^2^=0.811.

We then focused on the relationship between polyX content and tail dimensions. We calculated the total number of aas that make up all polyX regions within each TMP and plotted this value against the experimentally measured tail length for each phage (Fig. 5). The analysis shows a strong positive linear correlation (*R*²=0.811, *P*=1.56×10^−^⁴) between the total aa contribution from polyX regions and the final phage tail length.

This result from an independent, experimentally verified dataset confirms that the abundance of polyX regions, specifically the total number of aas they cover, is a strong predictor of the phage tail length. The low number of phages for which we could obtain experimental measures of their tail length limits the strength of our analysis, but the consistency of the trend across unrelated phages supports a real biological link between polyX expansion and tail size.

## Discussion

Here, it is shown that nearly every annotated TMP of tailed phages (95.53% of them) contains at least one polyX region. Given the established role of TMPs as molecular ‘rulers’ that determine tail length during virion assembly [38, 39], this enrichment invites a reconsideration of how length regulation operates. Earlier explanations have focused on tandem [14] or cryptic [6] repeats as the adjustable component in this system. Yet our study offers a refinement of that framework: polyX regions appear not as minor accessories but as a widespread mechanism that modulates the final dimensions of the phage tail.

When comparing both sequence types, polyX regions occurred far more frequently than TRs in TMPs (95.53% vs 52.47%), and notably, nearly half of the TMPs with polyX regions had no detectable TR at all. That absence is not trivial, as it establishes polyX sequences as an autonomous feature rather than a by-product of other repeat patterns. Their irregular spacing across proteins reinforces this view, since a regular, periodic arrangement would have suggested derivation from a TR. Even so, the two features are not mutually exclusive; proteins carrying multiple TRs tend to include polyX regions more often. Still, the overall picture places polyX as the more pervasive component of TMP architecture. However, in phages with the longest tails, such as those of the Siphovirus, polyX regions alone do not seem to be enough. These proteins often combine polyX with TRs. In this view, polyX provides the flexible baseline for elongation, while TRs add ordered repetition that maintains integrity in longer tails.

The composition of these low-complexity regions provides clues about their likely function. PolyA regions alone account for almost half (49%) of all polyX regions in the dataset. Such regions are recognized as hotspots for replication and transcriptional slippage, processes well documented in eukaryotes and prokaryotes [40–42], also within polyA contexts [32, 43]. The dominance of polyA and polyG regions in our sequences fits with this known biological pattern. It seems reasonable to interpret the polyA enrichment as a by-product of selective pressure favouring slippage-prone motifs, which in turn could facilitate adjustable protein length. This idea was previously hinted by Mahony and colleagues [6] with TRs as the responsible element for the tail length variation, which has proved in our study not to be exact.

That mechanism carries both evolutionary and structural consequences. On one side, slippage provides a rapid means for generating length diversity, potentially allowing phages to fine-tune tail morphology in response to changes in the host envelope or external environment. On the other, the aas most common in these regions (A, G, S and T) have small side chains that give flexibility to the polypeptide backbone. These stretches likely act as low-complexity hinges or soft joints within the TMPs. They may be crucial during tail assembly or infection, particularly for phages that use the bacterial pilus or flagellum as an adhesion platform. In those cases, the tail must coil around the filament before DNA injection, and a flexible protein scaffold would facilitate that motion. We hypothesize that polyX length variation, generated through replication slippage, provides phages with a low-risk, reading-frame-preserving mechanism to adjust TMP (and therefore tail) length in response to host envelope thickness or other physiological constraints, complementing the coarser length changes achievable through gain or loss of TRs. Protein length modulation through replication slippage is a common process in eukaryotes that has also been observed in prokaryotes and phages [44–46].

These two functional dimensions (slippage-based adaptability and mechanical elasticity) seem to converge on a shared purpose: maintaining the evolutionary and structural versatility of the phage tail. The strong positive correlation we observed between the total aa count in polyX regions and several experimentally measured tail lengths in our validation set supports this view. Even so, the correlation should not be overstated. The dataset used for validation was small (*n*=11 phages) and, for some phages, the experimentally measured tail length (obtained from cryo-Electron Microscopy (EM) or Transmission Electron Microscopy (TEM) studies in the literature) and the TMP aa sequence (obtained from the corresponding UniProt entry) may not have been derived from the exact same phage isolate but from different strains or subspecies of the same phage. Intraspecies length diversity has been described for polyA regions in humans [47], which opens the possibility that there may also exist polyA length diversity in different phages for a given species.

Beyond viruses, homorepeats (particularly polyA and polyQ) are common and studied in eukaryotic proteomes. They often localize within intrinsically disordered regions and contribute to protein–protein interactions, subcellular localization, regulation and adaptive flexibility [23]. In vertebrates and other animals, polyA occurs in hundreds of proteins, despite the risk that expansions can cause disease, suggesting that they confer functional advantages [48, 49]. Indeed, some polyA-containing human proteins are essential, evolve rapidly, and mediate cross-talk across pathways. The prevalence of polyA in phage TMPs may reflect a similar evolutionary selection process: alanine’s small side chain and codon properties may offer a good balance between structural flexibility and genetic evolvability. In eukaryotes, polyA coding sequences are often GCN nucleotide repeats, which favour slippage and polymorphism [49]. This could make polyA especially suited for regions where fine-tuned length variation matters, like TMPs.

Altogether, polyA prevalence across taxa supports the idea that polyA (and polyX) are not phage-specific but part of a general evolutionary toolkit: simple aa repeats with flexibility, slippage propensity and potential to modulate structure, interactions or stability. In that sense, polyX explains the TMP repeat signature, both in frequency and variability, more broadly than TRs alone.

## Supplementary material

10.1099/jgv.0.002341 Supplementary Material 1.

## Abbreviations

BH: Benjamini–Hochberg
HMM: Hidden Markov Model
pVOG: phage Viral Orthologous Group
TMP: tape measure protein
TR: tandem repeat
